# The Scientific Use of the Nosodes

**Published:** 1881-11

**Authors:** E. W. Berridge

**Affiliations:** London


### THE SCIENTIFIC USE OF THE NOSODES.

E. W. Berridge, M. D., London.

In that dim, mystical, prehistoric epoch known as "Once upon a time," two knights, travelling in opposite directions through a forest, came simultaneously upon a shield suspended from the branch of a tree. Thereupon they stopped to examine it, and began to discuss what it might mean. At length one of the knights remarked that it was made of gold, and the other replied that it was made of silver. They began to dispute about this, and presently came to blows. Some hours afterwards a third knight came up, and finding them both lying on the ground, wounded and unable to rise, inquired the cause. Having heard what had occurred, he informed them that they were both right in their respective assertions as to the composition of the shield, but that each was wrong in maintaining that he alone was right, *for the shield had two different sides*, one of gold and the other of silver. Whereupon the two combatants, the stranger having bound up their wounds and assisted them to rise and mount, rode away, each a sadder and a wiser knight.

This instructive legend has just been illustrated in its first stages by two of the best and bravest of our chevaliers, Drs. S. Swan and P. P. Wells, and to prevent the final catastrophe, rather than to criticise their writings, I venture to make these few remarks, commenting first on Dr. Wells' paper on "Unproved Remedies" in the August number of The Homœopathic Physician, and afterwards analyzing Dr. Swan's "Generalizations."

Dr. Wells regrets that the declaration of the International Hahnemannian Association did not contain an additional clause to the effect that the "minimum dose of the dynamized drug should also be of a drug which has been proved upon the healthy organism, before it can be given for the cure of the sick, as required by our law."

This clause was not inserted, because what is true in it is already implied, and what is not implied is not true. Our second declaration reads thus: "Whereas, this clearly teaches that Homœopathy consists in the law of similars, etc." Now, the expression "similars" necessarily implies the existence of two distinct groups of phenomena which are similar to each other, and as the "law of similars" signifies that to cure the sick we must give remedies capable of producing similar symptoms, it implies that these similar symptoms must be first ascertained, and the first and most obvious way of ascertaining this is by proving substances on healthy persons.

But when Dr. Wells not only claims that provings on the healthy constitute the *basis* of our materia medica, but that no true Homœopathician can ever legitimately give a remedy for the cure of the sick which has not been proved exactly in this manner, I would respectfully suggest that he falls into a fatal error, and demands the adoption of a hard and fast line which is neither required by Hahnemann, nor practicable in the existing state of our art.

Doubtless, were our materia medica complete, were every medicinal substance exhaustively proved on healthy persons, nothing more would be required; but as matters at present stand, and as they must remain for many years to come, there are so many gaps in our pathogeneses, so many symptoms and conditions which we cannot find recorded, in all their fullness and with perfect exactitude, in our provings on the healthy, that we gladly avail ourselves of any light which can be thrown upon the case, either by symptoms obtained upon the sick, or by clinical experience.

*Symptoms obtained upon the sick.*—In Section 142 of the *Organon*, Hahnemann says: "But how some symptoms of the simple medicine employed for a curative purpose, can be discovered even amid the symptoms of the original malady in diseases, especially in those of a chronic character that usually remain unaltered, is a subject for the exercise of the higher order of inductive minds, and must be left solely to masters in observation." If we refer to Hahnemann's own provings, we find him carrying out into actual practice the doctrine which he here enunciates. Symptom 303 of *Platinum* (in Allen's Encyclopedia) reads: "Cramps and stitches in the indurated uterus." Here then is a symptom given by the Master himself as a genuine effect of *Platinum* which was produced not only on a diseased individual, but on a diseased organ. Drs. Ad. Lippe, B. Fincke and myself (and probably many others) have published symptoms obtained thus upon the sick, which subsequent clinical verifications proved to be both genuine and important.

*Clinical Symptoms.*—Professor T. F. Allen, in his "Critical Examination of Our Materia Medica," says: "Others again have added to their provings symptoms observed in patients who have recovered after the administration of a drug. The principle being that if a drug removes a symptom it could produce it. Such symptoms are found in the provings of Hahnemann." As an illustration of this I will quote symptom 2453 of *Lycopodium:* "It seems to have a beneficial effect upon the softening and curvature of the bones." Nor have the Master's closest adherents failed to perceive their value. Bœnninghausen, *whose repertory Hahnemann said he preferred to all others,* incorporated hundreds of clinical symptoms into his works. Lippe's "Text Book" and Hering's "Guiding Symptoms" are full of these precious pearls. What Hahnemannian does not know the value of *Calcarea* when the symptom "feeling as if he had damp stockings on" is present, or of *Sulphur* for the symptom "emptiness at stomach at 11 A. M." *Yet these valuable keynotes are only clinical, not pathogenetic.*

And if clinical symptoms are often of such immense value, do we act wisely if we reject experience, even with unproved remedies? However much we may censure empiricism, the accidental *results* of empiricism cannot be ignored. We do not reject a proving of *Strychnia* because the drug was taken with suicidal intent, or of *Cannabis Indica* because taken for purposes of intoxication; ought we then to reject real cures because the physician did not *consciously* follow our law of similars when he prescribed the remedy? I do not, it is true, admit the *dictum*, that what will cure a symptom will produce it, for there are many ways of curing, though only *one* which is both of universal application and the best. But if it is true (as I think it is) that rarely repeated non-perturbative doses of the highest potencies can only cure when the remedy is truly homœopathic to the case, then all such cures must necessarily demonstrate the real homœopathicity of the curative agent, and a carefully conducted curative proving on the sick must be equal to a pathogenetic proving on the healthy.

Dr. Wells next discusses a very important doctrine taught by Dr. Swan. The latter maintains that these nosodes are not "unknown" or "unproved," but that each is proved in every case of its corresponding disease, and that the symptoms of these pathological conditions are well known. "It has been said," writes Dr. Wells, "that the disease giving us this potentized product is a proving of the product, and of a kind the most perfect. At the first glance this certainly has a seeming of truth on the face of it." Dr. Wells further admits that "disease producing this potentized agent is the result of a specific poison," and that "the poison is present in the product." But he demurs to the conclusion that "the phenomena of the disease are the expression of the action of the poison on the living organism, and therefore we have in them the most perfect of all provings." It certainly appears to me that if the truth of the premises be admitted, the conclusion must be true also.

Dr. Wells' objections are threefold. "We require," says he, "first of all, that the prover shall be in sound health at the time he takes the drug; and second, that the resulting phenomena be recorded at the time of their occurrence, with all their concomitants and modalities, with utmost exactness and detail, and this to the end of the prover's suffering;" and further, that we require "many repititions of this."

To the first objection I have already replied; to the second and third I answer that it is done daily in the practice of all careful homœopathic physicians who take copious notes of all the cases they treat, and has been done in the case of those enterprising French physicians who, when perfectly healthy, inoculated themselves with the syphilitic virus, and kept an accurate record of the result.

Dr. Wells doubts whether any reliable record of the symptoms of the patient from whom the nosode *Syphilinum* was taken has been made. Perhaps not. I do not know. But why should Dr. Wells, on that account, stigmatize it as an "unproved substance." Why does he say: "If not, then we have no proving of this product in the unrecorded phenomena developed in the life of the poisoned individual?" *Syphilinum has been proved;* aye, and proved on healthy persons in the highest potencies, too. So have other of the nosodes, the records of which are now lying before me. It is a fatal error to conclude that a remedy is "unproved" merely because it is not in the "Encyclopedia."

Dr. Wells' next objection is an important one. He says: "Even if there be such a record of any given case of the disease, and a portion of its poisonous product has been potentized, this record can only be available as a guide for the clinical use of this nosode as obtained from this particular case. The record of any other case, if made, may so differ from this, because of constitutional or circumstantial pecularities, as to render its product so unlike that of the first record as to render it wholly unsuitable as a guide for the clinical use of this. The same is true of the product of any other example of the disease."

Now, to quote Dr. Wells' own words: "At first glance this certainly has a seeming of truth on the face of it, and as truth is all we want we will see how this claim will bear examination." I held the same opinion myself at one time, and expressed that view—at page 468 of vol. 2 of *The Organon.* I now believe myself to have been mistaken. But, even supposing it is true, *what is there to prevent all Homœopathicians, using potencies prepared from the same original source*."

But is it really true? Granted, that the effects of syphilis on different persons have in each case peculiarities of their own. Does this invalidate their reliability? If we give a hundred different healthy persons the same dose of the same potency of the same preparation of the same drug, shall we not also find differences as well as resemblances in the resulting symptoms? The only possible source of error is that the virus itself, with which the proving is made, may not be free from admixture, and that, therefore, the resultant symptoms of the contagion may not be purely syphilitic. But when we put this matter to the test of actual experience, what do we find? Simply this, that *syphilis produces syphilis and nothing else*. We do not find that a cancerous or tuberculous patient who communicates syphilis communicates cancer or tubercule by the same act. Further, the clinical test, so far as investigations have been carried up to the present time, entirely confirms this view. Dr. Swan uses two preparations of *Syphilinum*, marked H and S (these marks do not mean *hard* and *soft*, as has been supposed, but are the initials of the physicians who procured them), and he has informed me that he sees no difference in their action. I have used two preparations, one obtained from Dr. Swan, and the other Jenichen's preparation, potentized higher, and I have found them both reliable when prescribed according to the symptoms of the proving. Whether this has been noticed with different preparations of other nosodes, I am unable to say, but the experience of other physicians on this point would be welcome. I conclude, therefore (subject to the result of further experimentation), that one specimen of the nosode of a well-defined pathological condition, such as syphilis, glanders, hydrophobia, tuberculosis, or scirrhus, will manifest the same curative and pathogenetic effects as another, not differing more than two or more specimens of *Belladonna*, which are and have been used indiscriminately both for provings and cures, even though one may not contain exactly the same percentage of *Atropine* as another.

Dr. Wells concludes his remarks on the nosodes by stating that "the further we pursue the matter of this class of unproved, so-called remedies, the more and greater difficulties we meet in the way of their intelligent incorporation into rational, sound, homœopathic practice. Indeed, as at present informed, this seems wholly impracticable." Believing as I do—and in this point I am in fullest harmony with Dr. Wells — that our law of similars is absolute, exclusive and universal, needing no "auxiliary or supplementary principles" whatever, I maintain that not only Dr. Swan's doctrine of the nosodes, but his two other generalizations also, are in complete accord with that law, *when they are strictly and logically interpreted*—nay, are indeed a very part of it.

Dr. Swan maintains the truth of these three generalizations:

1. Morbific matter will cure the disease which produced it, if given in very high potencies, and to any other than the person from whom it was obtained.

2. Any article of food that disagrees with a person, if given in a very high potency, will remove the symptoms and enable him to take the food with impunity.

3. A very high potency of a medicine will remove the pathogenetic effects of a much lower potency, or of the crude drug.

I have formulated these three generalizations, not exactly as they originally appeared in print, but so as to more clearly and fully express Dr. Swan's meaning, as frequently conveyed to me by conversation and correspondence. Also, though Dr. Wells' essay treats almost entirely of the nosodes, I have classed them together, not only on account of their interdependence, but also because an objection to the second generalization is made by Dr. Ad. Lippe, at page 365 of The Homœopathic Physician, and to the third generalization by Dr. C. Lippe, at page 94 of the same journal.

Before proceeding further, I will briefly comment on the last clause of the first generalization, "to any other than the person from whom it was obtained." It is remarkable that Hering's early experience was the direct opposite of this, for he then maintained that a nosode acted best when administered to the very patient who supplied it. Still more remarkable, in 1880, when Dr. Hering informed me orally that Dr. Swan was right, and that subsequent failures had led him to abandon his former doctrine as untenable. Dr. Deschere published in the *North American Journal of Homœopathy*, August, 1880, pp. 96–8, a remarkable case of pemphigus, cured by the highly potentized serum of its bullæ, *after five of the best indicated remedies had failed.* When we find two close observers flatly contradicting each other's experience, and a third appealing to his experience first in support of one view and then in support of the opposite, it is evidence that, while the facts cannot be questioned, the theories based upon them, in order to account for them, must be erroneous. My own opinion is that the source of the nosode matters nothing. Efficacy or failure to cure depends entirely on another consideration, as will be apparent later on.

I will now proceed to examine these three generalizations. As our law of similars admits no other, if true, they must be brought under it. As it admits no exceptions, they must invariably prove true, if true at all. But the curious feature of the case is that there are discrepancies in the evidence for and against these generalizations. While some aver that they have verified them, others on the other hand have failed to achieve success under their guidance. We are in fact in this dilemma, that while there are too many verifications to allow us to absolutely reject them, there are too many failures to allow us to unqualifiedly accept them. Bearing in mind the nature of law, it is evident that either the verifications are illusive and belong to some other law, or the exceptions are merely apparent and so prove the law.

Dr. Swan has mentioned to me one possible source of error: namely, that failure to cure may be from too low a potency; and as an illustration of this he reported to me a most malignant case of smallpox, where *Variolinum* MM (Swan) saved the patient's life, after CM (Swan) had utterly failed. Now this objection is perfectly valid in many cases, but it will not account for all, *because the very potencies which have failed to cure some cases have proved efficacious in others.* I have seen *Syphilinum* DM (F. C.) fail to cure a case of syphilis, but *Syphilinum* 1 M cured a case reported at p. 120 of THE HOMŒOPATHIC PHYSICIAN. I have seen *Croton* CM (Fincke) fail to cure a horse, which was poisoned by the large doses thereof recommended in a "Homœopathic" (! ?) Veterinary Manual, yet so low a potency of *Nux* as the 6th has cured an acute case of *Strychnia* poisoning. I gave a dose of *Saccharum officinale* 30 M (Fincke) to a man with whom sugar always disagreed, but without the slightest effect; yet Dr. Swan cured a case with the 5 M, 10 M and 41 M potencies. Clearly, therefore, this explanation will not account for *all* failures.

Nor, if we resort to the second fount of Homœopathic knowledge, do we find much light, for the reason that when Hahnemann wrote the fifth edition of the *Organon*, he was only just entering on the investigation of the subject. He says nothing about the power of a highly-potentized food to permanently antidote the idiosyncratic poisonous effects of that food. That he knew not that a high potency of a drug would antidote the effects of a lower or of the crude substance, is demonstrated by the note to section 246, where he states that in cases where *Sulphur* has been abused, it must be antidoted by *Mercurius* before the system will bear the action of even the 30th potency of *Sulphur*. Of the nosodes even he says but little, but that little is important and instructive, and in my opinion fully justifies their use within the limits of our law. While the Master in the Introduction to the *Organon* repudiates Lux's system of Isopathy, partly because impracticable, partly because the illustrations thereof are either not isopathic but honœopathic, or else rest upon insufficient evidence; in the note to section 56 he says: "A fourth mode of employing medicines in diseases has been attempted to be created by means of isopathy, as it is called; that is to say, a method of curing a given disease by the same contagious principle that produces it. But even granting this could be done, WHICH WOULD CERTAINLY BE A MOST VALUABLE DISCOVERY, yet after all, seeing that the miasm is given to the patient highly dynamized, and thereby, consequently, to a certain degree in an altered condition, the cure is effected only by opposing a *simillimum* to a *simillimum*." Here Hahnemann distinctly states that to give a nosode for the cure of the corresponding disease would not be isopathy, but Homœopathy, and, if practicable, would be a very valuable discovery. And, by analogy, this confirmation and endorsement of Dr. Swan's first generalization is a confirmation and endorsement of the other two.

But how does he teach that this "valuable discovery" should be carried into actual practice? By prescribing for the name of the ailment? By giving the nosode in *every* case of its corresponding disease? By no means! On the contrary, though at the conclusion of the introduction to the *Chronic Diseases* he speaks of *Psorinum* as homœopathic, and not isopathic, to psora, so far from implying it was indicated in *every* case, he follows this statement with the voluminous provings of no less than 49 antipsorics, which would have been useless had *Psorinum* been all-sufficient. Furthermore, though he enumerates nearly 500 symptoms of psora, he also declares that none of the nosodes, not even *Psorinum*, had up to that time been sufficiently proved to be used homœopathically, or to find a place among the pathogeneses of remedies especially adapted for chronic diseases.

This leads us to the doctrine maintained by Dr. Swan, but repudiated by Dr. Wells, that the nosode is really proved in each case of the disease. Hahnemann's teaching on this point has only a negative value. His rejection of the 500 symptoms of psora as a proving of *Psorinum* may simply mean that psora is a widely generic term, embracing many pathological conditions. Again, his non-acceptance, as provings, of the symptoms which he gives as indicating syphilis and sycosis, may be accounted for by the fact that they are too few in number, and too little defined by conditions and modalities to prove trustworthy indications for treatment, though reliable for diagnosis.

But another authority, our lamented Hering, has spoken plainly on this point, and his evidence is the more valuable as he at one time held an opposite opinion. In the *North American Journal of Homœopathy* for August, 1877, Dr. Hering wrote an article on "Our Nosodes," in which he put forth the following theory: "Every contagion acts in an entirely different way if communicated to the circulating fluids than if taken internally. If inoculated, or if given by subcutaneous injection, like the snake-bite or the bite of a dog, they act without exception as ferments; thus by zymosis. But when taken internally by the alcoholic extract; containing some salts so far chemically unknown, or some similar chemical combinations, it then acts like every other poison or drug. Dr. Wells does not acknowledge this, as it were, polar difference. He even calls infection by contagion a 'proving,' which is entirely wrong. No one would call a child's vaccination a proving of cow-pox."

In the November number of the same journal, I replied to this assertion, and after raising some theoretical objections to the doctrine of zymosis, I urged that the symptoms produced by the crude virus and the dynamized nosode must be of the same order, because, first, they produce many similar symptoms; second, the dynamized nosodes have cured symptoms as yet produced only by the crude virus; and third, diseases have been cured by their own highly dynamized nosode.

Soon after my letter appeared in print, Hering wrote to me promising a reply; but the reply never appeared. He had changed his views on the subject, and in his monograph of *Lyssin*, subsequently published, be incorporated the symptoms from the bite of the dog; while in his *Guiding Symptoms*, under the *Anthracinum* he says: "All symptoms produced by the poison on men are inserted, because the symptoms from the snake-bite and from the bee-sting have proved to be useful in numerous cases." But I remember asking Hering if he would incorporate the symptoms of syphilis in a proving of *Syphilinum*; and he replied, "No, not syphilis; it is too complicated."

Here then we have the solution of the whole problem in a nutshell. Every nosode is really proved in every case of the disease, *but we must carefully distinguish between the symptoms of the patient suffering from the disease, and the symptoms of the disease itself*, and none but "masters in observation" can be trusted to perform this difficult task, and even then the selected symptoms need clinical verifications before we can unreservedly accept them. It seems probable that the more acute and self-limited the disease, the less possibility there is of admixture with symptoms from other sources; and I have noticed that the earlier the stage of the disease, the more efficacious the nosode seemed to be, complications from bad treatment, or aroused latent dyscrasias not having been yet developed.

Since, therefore, the symptoms of the disease, when they can be accurately diagnosed and selected, are of the same order and nature as those produced by its dynamized nosode, it follows that the nosode must be a *simillimum* to the disease. But since it is also true that the symptoms of the disease are often complicated with those of other dyscrasias, or by bad treatment, it follows that in such cases the administration of the nosode will fail, because it is a *simillimum* only to a portion of the totality of the symptoms.

This reasoning also applies to Dr. Swan's second generalization. If the toxical effect of an article of food is merely an idiosyncrasy of the individual, a high potency of the same will permanently cure, as has often been done; but if it is merely a single symptom of a general morbid state, it will not cure, unless it happen to correspond also to the other symptoms. Under "aggravation from sugar," C. Lippe's excellent repertory gives eight remedies (to which *Medorrhinum* may be added); these remedies represent eight different dynamic changes from health, and, as no remedy can entirely fill the place of another, it is impossible that a high potency of *Saccharum* can cure every one of them.

Lastly, Dr. Swan's third generalization receives some light from the same argument. If the symptoms are *entirely* the result of the drug, then a much higher potency of the same drug will remove them; but if they are complicated with a previous morbid state, or if latent taint in the system has been aroused by them, the true antidote is one which corresponds to the totality of *both groups* of symptoms. Let me add here, that in the case of exceedingly sensitive provers, the higher potency, though it may antidote some of the effects of the lower, may also itself develope a train of severe symptoms. This I think I have detected in some provings.

Thus Dr. Swan's generalizations are probably correct; *pro-*

*vided they are most rigidly interpreted*, and so the totality of the symptoms strictly observed; and when thus interpreted, they are seen to form a part of our unerring, universal, and all-sufficient law of healing by drugs, *Similia Similibus Curantur.*

It has occurred to me that a tabular statement of my experience with nosodes, compiled from my case-books, might be of use. When the symptoms of the sick have corresponded with those of the provings of the *potentized* nosodes, I have of course prescribed them as I should any other indicated remedy. These cases I have not tabulated, but only those in which I have given a nosode for the cure of the corresponding disease *without* such indications—which cases now extend over a period of six years. These cases are comparatively few, for I have carefully guarded myself against falling into the empirical and reckless practice of prescribing a nosode for the *name* of the disease, when the totality of the patient's symptoms plainly indicate some other remedy. I have only given them, apart from the guidings of the provings of the potencies, (1) in cases where the remedies apparently best indicated had failed, and (2) when the symptoms were so vague that no remedy could be selected as the *simillimum.* In these two classes of cases I have considered myself justified in giving them, and watching the result. Up to the present time, I have never yet succeeded in completely *curing* a disease with its nosode, but I rarely failed in *relieving* it. I have also observed that the earlier the disease was treated, the more efficacious was the nosode, complication not having had time to arise. Hence I conclude that a nosode must always be a *simile* to every case of its disease, but by no means always a *simillimum*, owing to these complications. Some cases I have omitted in the following table, not having received any further report.

TABULAR STATEMENT.

(1) *Medorrhinum* in gonorrhœa. Given in six cases. In three the symptoms were relieved but not cured by it. In one they were all removed, but reproduced apparently by a dose of *Sulphur* 2 M which the patient took on his own account; the new symptoms plainly indicated *Cantharis.* In one it did nothing, because the patient took *Thuya* 30 the next day, without my knowledge. In one it increased and developed symptoms which were then met by their appropriate *simillimum.* (This last patient left me because I would not reduce my fees to him.)

(2) *Syphilinum* in syphilis. Three cases; all relieved, but not cured by it.

(3) *Scirrhinum mammæ*. Relieved temporarily two fatal cases, one of scirrhus of the breast, and the other of the uterus. Also relieved a fibrous tumor of breast (no absolute proof of scirrhus), but the patient, being better, ceased treatment. Dr. Swan informs me that he sees no difference between the action of his *Scirrhinum mammæ* and *faciei*, and the former has acted equally well in my hands in *scirrhus mammæ* and *uteri*.

(4) *Hydrophobinum* in hydrophobia. Case reported in THE HOMŒOPATHIC PHYSICIAN for July. Dr. Swan thinks a higher potency would have saved the boy; perhaps so, but I think he was completely worn out when I saw him. The next case shall have a higher potency, and I hope will send earlier and not waste precious hours.

(5) *Psorinum* in scabies. Some cases (number unrecorded) in one family relieved, but other remedies also needed.

(6) *Variolinum* in *severe* variola. Three cases. No apparent effect; one died, and the other two were pitted and had sequelæ. The potencies used were CM (Swan) and 300 (Jenichen). Higher potencies in other hands have, however, proved more efficacious.

(7) *Tuberculinum* in tubercular phthisis. Two cases, one chronic and the other acute. Gave CM (Swan) in each case. The first is a doubtful case, because in two or three days the symptoms changed and clearly indicated *Arnica* which was given. In the second neither this remedy nor the remedies prescribed by other physicians did any good. It was one of the most rapid cases on record.

(8) *Melitagrinum* in eczema. One case relieved but not cured by it; another case relieved and still under its action. (Two colleagues have *cured* cases with it.)

(9) *Asthmatos ciliaris* in catarrh. In the *Medical Advance* for September, Dr. Swan reports a rapid cure. Just after reading it I caught a cold. I can never cure my own catarrhs till they reach their second stage, for there is nothing characteristic about them. So on the principle *fiat experimentum in corpore* (the Apostle Paul speaks of our "vile body"), I took a dose of CMM (Swan). It did nothing, except perhaps to hurry it into its second stage, when *Bryonia* was indicated, and at once acted favorably.

(10) I have left the best to the last, that it may be remembered. A few weeks ago, a colleague asked me about a case of *psoriasis inveterata* which had been brought to him for treatment. He had not yet prescribed, being in doubt what to give. The patient had been under the care of a strict Hahnemannian of great experience, but without result. Hence my friend concluded that it was no use giving the old, apparently-indicated remedies, as they had doubtless been all given before. I agreed with him, and said that in such a case it would be lawful to use the nosode. He gave *Psoriasinum* 1500 (F. C.), *and with marvellous result.* The cure is not yet complete; it may prove temporary only, or other remedies may be needed, but this *fact* remains, that the despised, "unproved" nosode did more than the old, well-proved remedies, even in the hands of a master of the materia medica.*

* We are glad to publish the above paper of Dr. Berridge; the more so as we cannot but consider his arguments for the use of *unproved* nosodes and his "tabular statement" of their inefficiency, *as strong arguments against their use.* No better reason for *not* using them can be found than Dr. Berridge gives, when he admits: "Up to the present time I have never yet succeeded in completely *curing* a disease with its nosode, but rarely failed in *relieving* it." We are confident that the homœopathic profession will be slow to desert certain and reliable drugs for uncertain and confessedly unreliable medicinal agents.

We would add that, where Dr. Berridge fails to convince in his arguments, or to cure with his agents, others will be likely to *do no better.*—EDITOR.

